# Quantitative Serum Nuclear Magnetic Resonance Metabolomics in Large-Scale Epidemiology: A Primer on -Omic Technologies

**DOI:** 10.1093/aje/kwx016

**Published:** 2017-05-10

**Authors:** Peter Würtz, Antti J Kangas, Pasi Soininen, Debbie A Lawlor, George Davey Smith, Mika Ala-Korpela

**Keywords:** amino acids, biomarkers, drug development, fatty acids, Mendelian randomization, metabolomics, nuclear magnetic resonance, serum

## Abstract

Detailed metabolic profiling in large-scale epidemiologic studies has uncovered novel biomarkers for cardiometabolic diseases and clarified the molecular associations of established risk factors. A quantitative metabolomics platform based on nuclear magnetic resonance spectroscopy has found widespread use, already profiling over 400,000 blood samples. Over 200 metabolic measures are quantified per sample; in addition to many biomarkers routinely used in epidemiology, the method simultaneously provides fine-grained lipoprotein subclass profiling and quantification of circulating fatty acids, amino acids, gluconeogenesis-related metabolites, and many other molecules from multiple metabolic pathways. Here we focus on applications of magnetic resonance metabolomics for quantifying circulating biomarkers in large-scale epidemiology. We highlight the molecular characterization of risk factors, use of Mendelian randomization, and the key issues of study design and analyses of metabolic profiling for epidemiology. We also detail how integration of metabolic profiling data with genetics can enhance drug development. We discuss why quantitative metabolic profiling is becoming widespread in epidemiology and biobanking. Although large-scale applications of metabolic profiling are still novel, it seems likely that comprehensive biomarker data will contribute to etiologic understanding of various diseases and abilities to predict disease risks, with the potential to translate into multiple clinical settings.

Omics profiling—genomics, epigenetics, proteomics, and metabolomics—is becoming increasingly widespread in the worldwide efforts to prevent noncommunicable diseases. This is driven by the quest for better etiologic understanding but also importantly by technical developments that allow quantitative high-throughput methodologies for several -omics, notably genome-wide single nucleotide polymorphisms (1, 2), genome-wide DNA methylation (3, 4), and detailed metabolic profiling (5–14). Advancements in the experimental throughput of metabolomics have paved the way for applications in large-scale epidemiologic studies, and the utility of metabolic profiling to advance our understanding of disease etiologies and to improve risk predictions is becoming apparent (5, 15–18). The simultaneous quantification of circulating biomarkers across multiple pathways gives a fine-grained snapshot of a person's metabolic state and offers molecular insights on health and disease. Recent advancements in experimental throughput have paved the way for widespread applications of metabolic profiling in population cohorts, with an initial focus on the etiology and biomarkers of cardiometabolic diseases (5–7, 19–24).

General aspects of design and analysis of metabolomics studies in epidemiologic research has recently been reviewed in this journal (20). In this review, we focus on large-scale epidemiologic applications of nuclear magnetic resonance (NMR) metabolomics for quantifying circulating biomarkers. The emphasis is on a specific platform for quantitative high-throughput serum metabolomics, because this is the first, and currently the only, NMR-based metabolomics platform broadly applied in large-scale epidemiologic studies (5). We start out by covering the overall characteristics of this platform. We also detail how integration of metabolic profiling data with genetics can enhance drug development, and we provide some reflections on study design and statistical analyses based on lessons learned from various applications of metabolic profiling in large cohort studies.

## FROM LIPOPROTEIN QUANTIFICATION TO COMPREHENSIVE METABOLIC PROFILING

### A high-throughput NMR platform for epidemiologic applications

NMR-based lipoprotein quantification has a long history (22, 25, 26). During the past decade, our research team has focused on the development of a quantitative NMR metabolomics platform for high-throughput profiling of serum (and plasma) samples, in which the lipoprotein quantification has been taken to subclass level, and the metabolic quantifications extended broadly beyond lipoproteins (5, 23). The development has been made from the initial phase with applications in epidemiology and clinical settings in mind. This focus has directed experimental optimization for absolute quantification, maximal throughput, and cost-effectiveness.

In addition to analyses of lipoprotein measures, recently extensively reviewed by Mallol et al. (25), NMR spectroscopy can also be used as a general method to quantify multiple molecular constituents in serum and other biofluids (27–29). However, with the exception of studies using the NMR metabolomics platform described in this review, very few applications of NMR for quantifying a broad spectrum of circulating metabolites have been published in epidemiologic contexts (5, 29, 30). Thus, the situation in the field is currently such that the large-scale applications of serum NMR metabolomics (summarized in Table 1) include only studies based on the platform in question.

**Table 1.** Metabolic Profiling Studies That Had >5,000 Participants and That Used Quantitative Serum Nuclear Magnetic Resonance Metabolomics

Focus	Study Populations and Description	Reference(s)
*Biomarkers for Disease Events and Risk Prediction*		
Cardiovascular disease	Biomarker discovery for risk of first incident cardiovascular event (*n* = 13,441 Finns and Britons from 3 population-based studies with 12–23 years of follow-up). Phenylalanine and MUFAs were found to be predictive of higher CVD event risk, whereas omega-6 fatty acids and docosahexaenoic acid levels were inversely associated with CVD event risk, after adjustment for routine lipid levels and other risk factors. These 4 biomarkers in combination improved risk reclassification above established risk factors in 2 validation cohorts. Analytic and biological comparison of biomarkers quantified by the NMR platform and 2 MS platforms (*n* > 2,000 Americans and *n* > 2,000 Finns in the analytic and biological comparison between NMR and MS biomarker associations).	7
All-cause mortality	Discovery and replication of biomarkers for 5-year risk of death. Glycoprotein acetylation, albumin, VLDL particle size, and citrate were found to be strongly predictive of the short-term risk of all-cause mortality, and a biomarker score was shown to improve risk prediction and illustrate a potential clinical application for patient prioritization (*n* = 17,345 Estonians and Finns from 2 population-based studies).	37
Inflammation	Molecular etiology of glycoprotein acetylation, the strongest biomarker for all-cause mortality identified in Fischer et al. (37), investigated by cytokine panels and whole blood gene expression networks. Glycoprotein acetylation was found to be a marker of chronic inflammation related to increased neutrophil activity and strongly predictive of the long-term risk for sepsis and respiratory infections (*n* ≈ 10,000 Finns from 3 population-based cohorts).	16
Type 2 diabetes mellitus	Cross-sectional associations of 8 amino acids with glycemia (*n* ≈ 9,400 Finnish men) and risk for onset of type 2 diabetes (*n* = 526). Branched-chained and aromatic amino acids, as well as alanine and glutamine, were predictive of diabetes risk, partly explained by insulin resistance.	34, 36, 42, 43, 90
	Cross-sectional and prospective associations of the ketones acetoacetate and β-hydroxybutyrate (*n* ≈ 9,400 Finnish men/*n* ≈ 4,300 in prospective analyses), showing positive association with future glucose tolerance and, in the case of acetoacetate, higher risk for diabetes onset. The results were attributed to insulin sensitivity rather than insulin resistance.	56
	Associations of fatty acids with 5-year glucose tolerance and type 2 diabetes risk (*n* ≈ 9,400 Finnish men/*n* ≈ 4,300 in prospective analyses), showing that glycerol, triglycerides, and MUFAs are positively associated with diabetes risk, and omega-6 fatty acids are inversely associated.	34
	Prospective associations of lipids and lipoprotein subclass measures with glycemia and type 2 diabetes risk (*n* ≈ 6,607 Finnish men), showing stronger predictive associations of lipoprotein and apolipoprotein ratios than routine lipid measures.	78
*Metabolic Risk-Factor Characterization*		
Adiposity	Mendelian randomization analyses of body mass index as a causal risk factor for systemic metabolism: causal effects of adiposity on numerous metabolic measures, including branched-chain and aromatic amino acids, omega-6 fatty acids, and glycoprotein acetylation as well as multiple lipoprotein lipid subclasses and particle size measures (*n* = 12,664 young adults from 4 population-based cohorts in Finland). Weight changes were paralleled by extensive metabolic changes, with a similar metabolic signature as observed cross-sectionally and genetically (*n* ≈ 1,500 with 3 time points).	8
Insulin resistance	Cross-sectional associations of metabolites with insulin resistance index (*n* = 7,098 young Finnish adults from 2 population-based cohorts in Finland). Results showed numerous strong metabolite associations with insulin resistance, independent of components of the metabolic syndrome, and uncovered multiple sex-specific associations and adiposity interactions.	21
	Cross-sectional associations of lipoprotein subclass measures with different indices for insulin resistance, showing more prominent associations with liver insulin resistance than with whole-body insulin sensitivity (*n* = 8,750 Finnish men).	91
	Cross-sectional associations of lipoprotein subclass profiles with glucose tolerance categories and insulin resistance index, showing prominent associations of insulin sensitivity with VLDL and HDL subclasses, including heterogenic associations for small HDL (*n* = 9,400 Finnish men).	36
Sex hormone–binding globulin	Mendelian randomization analysis indicating that sex hormone–binding globulin is strongly associated with numerous circulating metabolites but not a causal risk factor for the systemic metabolic effects (*n* ≈ 16,000 from 4 Finnish cohorts for either cross-sectional or causality analyses).	74
Birth weight	Associations of lower birth weight with the metabolic profile in adolescents and adults. The metabolic associations found were of modest magnitude and displayed a similar overall metabolic signature as the metabolite association pattern with higher adiposity (*n* = 18,288 from 7 population-based cohorts from Finland and the United Kingdom).	73
Menopause and aging	Associations of age, sex, and menopause with the systemic metabolic profile, assessed cross-sectionally (*n* ≈ 23,000 people from 8 cohorts in Finland and Estonia). Menopause status was associated with glutamine, tyrosine, and isoleucine, along with atherogenic lipoprotein measures.	92
Alcohol consumption	Cross-sectional associations of alcohol consumption with the systemic metabolic profile (*n* = 9,778 young Finnish adults from 3 population-based cohorts). Results showed robust biomarkers for alcohol intake beyond routine lipids, including adverse associations with omega-6 fatty acids, MUFAs, glutamine, and citrate. Longitudinal analyses showed that the metabolic signature of alcohol intake track with changes in alcohol intake (*n* ≈ 1,450 with 3 time points).	93
Vitamin D	Cross-sectional associations of serum 25-hydroxyvitamin D concentrations with the systemic metabolic profile (*n* = 1,726 in a discovery cohort and *n* = 6,759 in a replication cohort). Results showed 30 replicated metabolic associations, including constituents of large VLDL and small LDL subclasses and related measures such serum triglycerides, as well as fatty acids and measures reflecting the degree of fatty acid saturation.	54
*Metabolic Effects of Drug Interventions*		
Statin therapy	Effects of statins on the systemic metabolic profile, assessed for 4 longitudinal cohorts (*n* = 5,590 with 2 time points). Statins were shown to lower small VLDL particles and remnant cholesterol, in addition to the LDL-lowering effects. Minimal or no side effects on nonlipid metabolites were observed. The observational results were validated by Mendelian randomization analyses in 8 population-based cohorts (*n* = 27,914), with associations in the *HMGCR* gene perfectly matching the longitudinal associations.	13
Hormonal contraceptives	Effects of hormonal contraception on the systemic metabolic profile assessed in cross-sectional and longitudinal settings (*n* = 5,841 women from 3 Finnish cohorts; *n* = 869 with 2 time points). Combined oral contraceptive pills were shown to have very prominent metabolic effects, including changes in many fatty acids and amino acids, and predominantly related to higher cardiometabolic risk. The metabolic aberrations were reversed upon discontinuation. Progestin-only contraceptives had little effect on systemic metabolism.	75
*Genome-Wide Association Studies*		
Genetic determinants of circulating biomarkers	GWAS of 115 metabolic measures and 99 derived measures from the NMR platform. The study identified metabolic associations at 31 loci, including 11 novel loci (*n* = 8,330 individuals from 5 population-based cohorts in Finland), and provided heritability estimates from twin pairs (*n* = 561 pairs; 221 monozygotic and 340 dizygotic pairs).	85
	GWAS of 123 metabolic measures from NMR metabolomics (up to *n* = 24,925 individuals from 14 European cohorts). The study identified associations at 62 loci, including 8 novel loci for amino acids and other metabolites. The results further elucidated the effects of lipoprotein(a) on lipid metabolism.	30
	GWAS of 11 metabolic networks, identifying 34 genomic loci, of which 7 were novel. The results illustrate how multivariate analysis of correlated metabolic measures can boost power for gene discovery (*n* = 6,608 from 2 Finnish cohorts).	94
*Functional Genetics*		
Lipid genes	Metabolic profiling and genetic fine-mapping of 95 lipid loci, showing refined lipid associations with numerous loci and illustrating how most lipid genes affect a broad span of lipid measures (*n* = 8,330 individuals from 5 population-based cohorts in Finland).	83
Lipid genes/pleiotropy	Assessment of pleiotropy in 6 cholesterol- and triglyceride-related genes. The broad lipid association patterns indicated that the lipid loci cannot be attributed to a single routine lipid measure, and the implications for Mendelian randomization studies are discussed (*n* = 10,547 individuals from 3 population-based cohorts in Finland).	51
Type 2 diabetes genes	Lipoprotein subclass profiling of 34 risk loci for type 2 diabetes. The results suggest that only a small number of diabetes loci affect lipoprotein lipid measures (*n* = 6,580 individuals from a population-based cohort of Finnish men).	95
Liver function genes	Metabolic profiling of 42 genetic loci associated with concentrations of liver enzymes in plasma, highlighting multimetabolic effects of several loci (*n* = 6,516 individuals from 2 population-based cohorts from Finland and the United Kingdom).	96
Blood-pressure genes	Metabolic profiling of 29 blood pressure genes, indicating weak (if any) effects of blood pressure on the circulating metabolic measures (*n* = 7,032 individuals from 3 population-based cohorts in Finland).	97
Interleukin-1 inhibition gene	Lipoprotein subclass profiling of genes encoding IL-1 receptor antagonist, detailing the proatherogenic lipid effects of IL-1 inhibition, with implications for treatment of cardiometabolic disease by IL-1 inhibitors (*n* = 8,330 individuals from 5 population-based cohorts in Finland).	76
Triglyceride metabolism gene	Metabolic profiling of a rare variant in *APOC3*, detailing the VLDL effects of *APOC3* and showing partly independent effects compared with the *LPL* gene (*n* = 13,285 from 2 population-based cohorts in the United Kingdom).	67
HDL metabolism gene	Lipoprotein subclass profiling and genetic fine-mapping of *GALNT2*, a locus associated with HDL cholesterol. Results showed the most prominent associations of *GALNT2* with cholesterol in medium-sized HDL particles (*n* ≈ 10,000 Finnish men).	98
*Bioinformatics Applications*		
Multivariate meta-analysis of genome-wide studies	Multivariate associations of lipoprotein subclass measures (and genotypes), similar to the approach used in Inouye et al. (94), but allowing analysis based on summary statistics-based of single or multiple cohorts (*n* = 10,753 from 3 Finnish population-based cohorts).	99
Multivariate gene-metabolome associations	Bayesian reduced-rank regression to assess the impact of multiple single nucleotide polymorphisms on a high-dimensional phenotype, demonstrated for the case of lipoprotein subclass measures. Two novel lipid genes were identified by the multivariate GWAS approach (*n* ≈ 10,000 from Finnish 3 population-based cohorts).	100
Multiple output regression with latent noise	Study illustrating how structured noise can, and should, be taken advantage of when assessing the associations between covariates and target variables, using multi-omics data and various metabolic measures (*n* = 5,211 from 2 Finnish population-based cohorts).	101
Network analysis integrating genome and metabolome	Methodology to assess differences in molecular associations and underlying genetic variants, illustrated in the context of obesity (*n* = 7,255 from 2 Finnish population-based cohorts).	102

Abbreviations: *APOC3*, apolipoprotein C3 gene; CVD, cardiovascular disease; *GALNT2*, UDP-*N*-acetyl-alpha-D-galactosamine: polypeptide *N*-acetylgalactosaminyltransferase 2 gene; GWAS, genome-wide association study; HDL, high-density lipoprotein; *HMGCR*, 3-hydroxy-3-methylglutaryl-coenzyme A reductase gene; LDL, low-density lipoprotein; *LPL*, lipoprotein lipase gene; MS, mass spectrometry; MUFA, monounsaturated fatty acid; NMR, nuclear magnetic resonance; VLDL, very low-density lipoprotein.

An overview of the metabolic biomarkers quantified by the high-throughput serum NMR platform is shown in Web Figure 1 (available at https://academic.oup.com/aje). A multitude of metabolic measures is quantified directly from serum in a single experiment. The profiling covers both standard lipid measures and a wealth of other metabolic biomarkers. In contrast to other NMR methodologies of advanced lipoprotein profiling (25, 31–33), this platform also provides quantification of many fatty-acid measures, some abundant proteins, and a broad range of low-molecular-weight metabolites together with very detailed lipoprotein subclass profiling (5). The panel of biomarkers has not been preselected based on anticipated biological relevance, but the metabolic measures are included because it is feasible to quantify these measures robustly in a single experiment (23). Circulating metabolites at concentrations down to ≈10 μmol/L are quantified, but the exact limit depends on the molecular identity. The biomarker output provided contains the majority of the metabolic information reliably quantifiable by NMR spectroscopy of serum (5, 23). Even with a substantial increase in the measurement time (at the expense of cost-effectiveness), only a few measures could be added to the biomarker panel. The experimental capacity is linearly scalable, dependent only on the number of spectrometers. The pricing for the entire biomarker panel is comparable to that of the more restricted lipid testing by routine clinical chemistry methods (5).

In the case of the NMR metabolomics platform discussed here, over 200 biochemically and metabolically distinct measures are given as the standard output (Web Figure 1). This number includes around 150 primary concentrations as well as selected ratios. For instance, individual fatty-acid concentrations relative to total fatty acids are included because they better reflect the biology of individual fatty acids than do the absolute concentrations (34), and the ratios are commonly the only metric captured by complementary analytic methods (35). The lipid composition measures of lipoprotein subclass particles are also included in the overall number of metabolic measures because they define a biologically separate entity of measures (36).

### Sample preparation

The blood samples routinely collected in epidemiologic cohorts and biobanks can be directly used for metabolic profiling. In general, any collection of blood samples amenable for lipid testing by standard methods can be used for the NMR platform. This means that samples stored long-term must have been kept at a temperature of −70°C or colder to retain the composition of lipoprotein particles, and the integrity of other metabolic measures. Both fasting and nonfasting samples can be analyzed (5, 37). The spectral characteristics of serum samples reflect various aspects of sample quality, and quality-control procedures can detect irregularities due to potential sample degradation. A sample volume of either 100 μL or 350 μL is used for the analysis, with both volumes yielding the same set of metabolic measures; analyses with the larger volume are more cost-effective due to shorter measurement time in the NMR spectrometer. One of the primary advantages of NMR is the minimal sample preparation required. Automated liquid handlers simply mix a buffer with the serum and move the material to 96-format racks of NMR tubes. The racks are subsequently inserted into the robotic sample changer, cooled to refrigerator temperature. The sample changer holds 480 samples simultaneously, yielding over 24 hours of automated measurements before the need to reload more samples. Automated shimming, accurate temperature control, and stable electronics in modern off-the-shelf NMR spectrometers have been a prerequisite for the high throughput. Details of the present platform have been described previously (5, 23). The original methodology was based on 3 molecular windows, of which 2 were acquired from the original serum samples, and 1 from the serum lipid extracts (23, 38, 39). More recently, a faster method has been developed in which the computational analysis circumvents the need for experimental lipid extractions.

In NMR spectroscopy, absolute quantification of metabolic measures in absolute units, rather than relative to another measure, can currently be achieved without external standards added to the blood specimen (40). In the NMR metabolomics platform that is the focus of this review, advanced proprietary software with integrated quality control is used to convert the spectral information to absolute concentrations of the metabolic measures. The basis for the metabolite quantification is Bayesian modeling, as described previously (5, 23, 41). The output data for each sample comprise a list of concentrations for the metabolic measures summarized in Web Figure 1. In comparison with clinical chemistry assays, the NMR metabolomics platform essentially just provides more biomarkers in a single experiment. The accuracy of biomarker quantification by the platform is comparable to what is commonly achieved by assays routinely used in clinical chemistry (see the caption for Web Figure 1) (30). The consistent biomarker quantification is due to the inherently reproducible nature of NMR spectroscopy; the samples never come into contact with the radiofrequency detector in the NMR spectrometer. This makes NMR metabolomics essentially free of batch effects that commonly hamper applications of mass spectrometry (MS) to large-scale epidemiologic studies. Biomarker quantification directly from serum, without any sample extraction procedures, further contributes to the high reproducibility. The NMR metabolomics platform featured here employs a targeted approach, meaning that an a priori defined set of metabolites is quantified from the experimentation. The platform is therefore not designed for novel biomarker discovery as such, in contrast to untargeted metabolomics approaches. Nonetheless, epidemiologic analyses based on the NMR platform have identified multiple novel and emerging biomarkers for cardiometabolic diseases (5, 7, 34, 42–44), because many of the quantified metabolic measures have not previously been studied in large cohorts. The pros and cons of targeted versus untargeted metabolomics approaches have been reviewed elsewhere (20, 45).

### NMR, MS, and clinical chemistry: analytic and biological consistency

**Figure 1.**
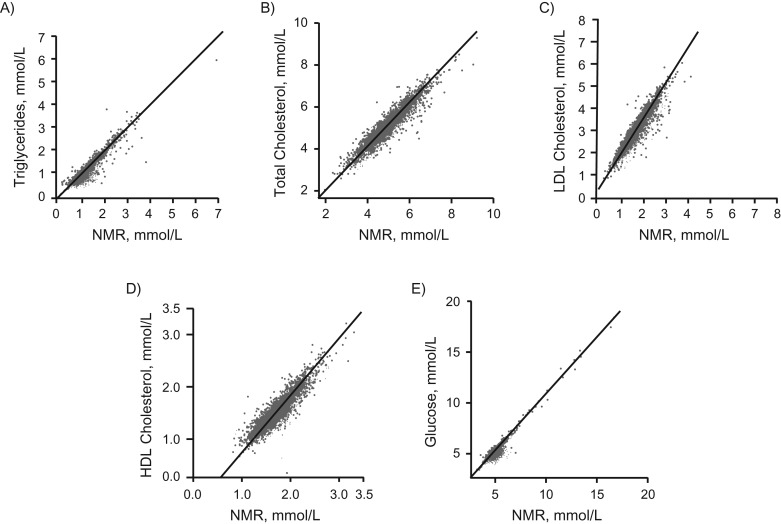
Comparison of lipoprotein lipid and glucose quantification in an epidemiologic setting, using nuclear magnetic resonance (NMR) (2013) and routine clinical chemistry assays (*y*-axis) (*n* = 2,749 from the Avon Longitudinal Study of Children and Parents (ALSPAC) Mothers Cohort) (103). The correlation coefficients are 0.95 (A), 0.94 (B), 0.93 (C), 0.91 (D), and 0.96 (E). The lower concentration of low-density lipoprotein (LDL) cholesterol quantified by NMR than by the Friedewald approximation stems from the latter also containing intermediate-density lipoprotein cholesterol (104). The NMR-based LDL cholesterol refers specifically to cholesterol in the LDL particles with the sizes as defined in Web Figure 1. The correspondence of these measures varies slightly from cohort to cohort, but the correspondence is generally excellent between the clinical chemistry and the NMR for these measures. It is important to note that the comparisons illustrated here do not show strict analytic comparisons with samples undergoing identical processing and storage time, but rather indicate analytic consistency demonstrated in epidemiologic settings. No quantitative assessment of analytic correspondences is therefore made here. When it comes to potential clinical applications of metabolic profiling, more analytic and clinical testing is required, particularly with those metabolic measures that are intended to be used as part of diagnostic protocols. It is also to be expected that official accreditations of analytic and laboratory procedures will be a prerequisite for widespread clinical applications. HDL, high-density lipoprotein.

**Figure 2.**
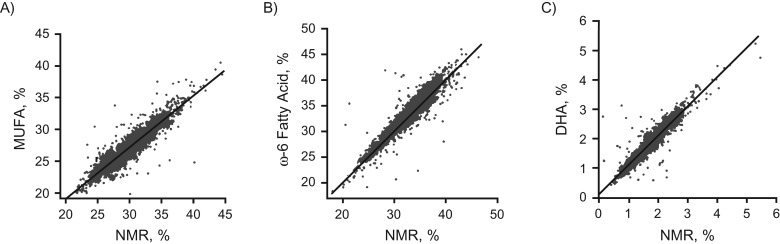
Comparison of circulating fatty-acid quantification in an epidemiologic setting, nuclear magnetic resonance (NMR) and gas chromatography (*y*-axis) (*n* = 2,193 from the Cardiovascular Risk in Young Finns Study) (7). The correlation coefficients are 0.92 (A), 0.94 (B), and 0.94 (C). See note on Figure 1 for the analytic correspondence. DHA, docosahexaenoic acid; MUFA, monounsaturated fatty acid.

**Figure 3.**
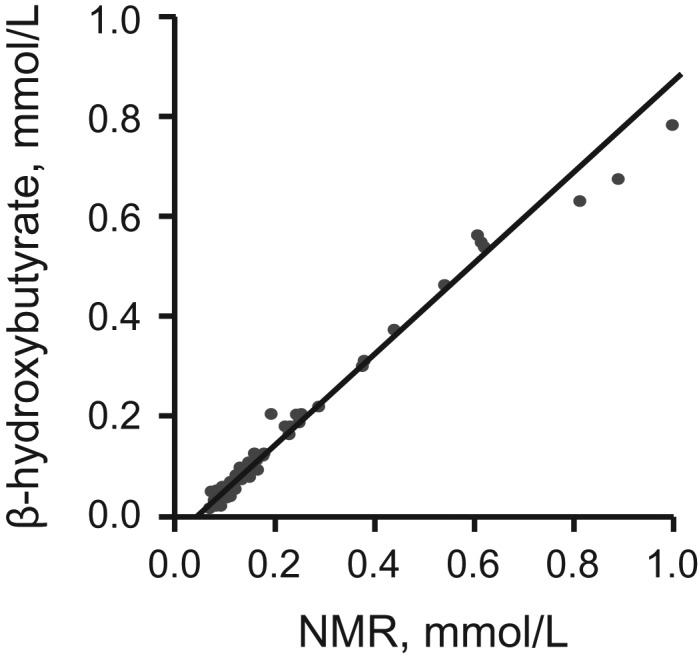
Comparison of circulating β-hydroxybutyrate quantification in an epidemiologic setting, using nuclear magnetic resonance (NMR) and an enzymatic method (*y*-axis) (*n* = 56) (105). The correlation coefficient is 0.98. See note on Figure 1 for the analytic correspondence.

**Figure 4.**
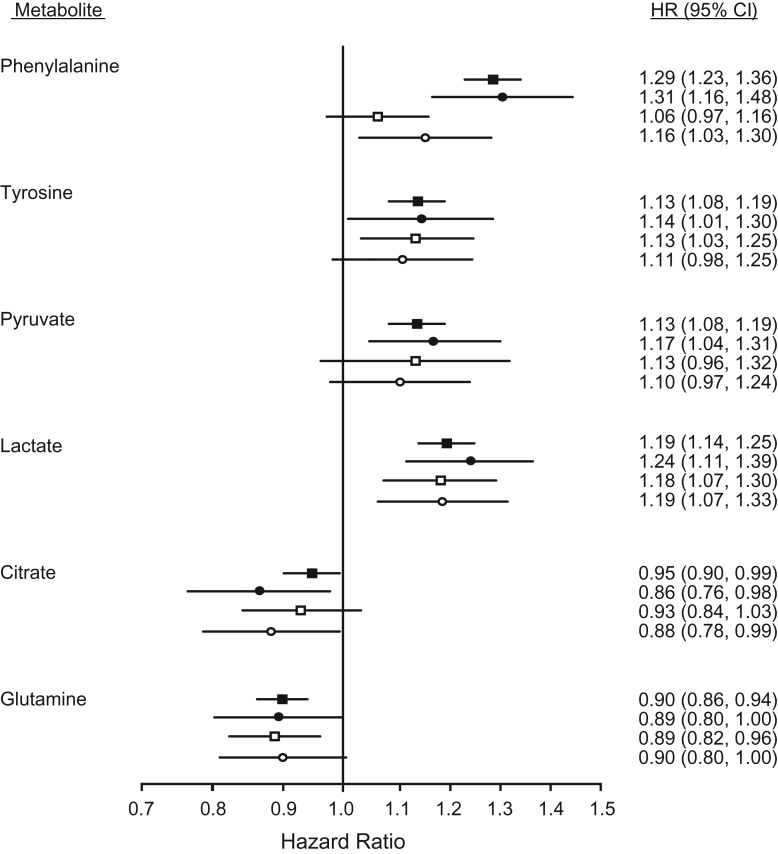
Biomarker associations with cardiovascular event risk for selected polar metabolites quantified by both nuclear magnetic resonance (NMR) and mass spectrometry (MS). Filled squares indicate hazard ratios for incident cardiovascular disease, adjusted for age and sex, for 13,441 individuals (1,741 events) profiled by NMR. Open squares show the same biomarker associations in the Framingham Offspring Study (2,289 individuals and 466 events) profiled by MS. Circles indicate the biomarker associations compared for the same subset of 679 individuals (305 events) profiled both by NMR (filled circles) and MS (open circles). The figure is adapted from Würtz et al. (7). CI, confidence interval; HR, hazard ratio.

Figure 1 shows that biomarker concentrations quantified by the NMR metabolomics platform were highly consistent with the concentrations obtained from routine clinical chemistry. Figures 2 and 3 show that quantification of emerging biomarkers, such as fatty acids and ketone bodies, by NMR is also coherent with results from other analytic methods. With quantitative biomarker data, it does not make a fundamental difference whether a metabolic measure is quantified by NMR or by alternative analytics—if each method identifies a particular molecular measure, only the accuracy and precision of the concentration measurement may differ (i.e., we do not have an NMR-molecule, an MS-molecule, or a clinical chemistry molecule but only a molecule). For applications of metabolomics in epidemiology, consistency of metabolic biomarker associations with disease events across different platforms is important, maybe more so than exact analytic correspondence in absolute concentrations. Figure 4 shows that emerging biomarkers quantified by both NMR spectroscopy and 2 widely used MS platforms have similar associations with disease incidence. These results suggest that associations of amino acids and gluconeogenesis metabolites with cardiovascular disease (CVD) risk are broadly consistent in their association with CVD, regardless of whether the biomarkers are quantified by NMR or MS. Accordingly, associations of amino acids with the risk for type 2 diabetes mellitus have also been consistent across NMR and MS platforms (46).

The advantages and disadvantages of NMR and MS—2 key technologies for metabolic profiling—have recently been extensively covered in multiple reviews (12, 45, 47–49). These analytic techniques offer mainly complementary insights, partly due to their different biomarker coverage. The higher sensitivity of MS allows for quantification of low-concentration metabolites and thus more in-depth characterization of pathophysiological mechanisms (9, 50). In contrast, the cost-effective metabolite quantification by NMR favors large-scale epidemiologic studies, such as profiling of entire cohorts and clinical trials (5, 7). For example, applications of Mendelian randomization for inferring causal relationships with genetic instruments are demanding in terms of sample-size requirements, and therefore they benefit from the high throughput and robust quantification offered by NMR (5, 13, 30, 51). The possibility with NMR to quantify biomarkers directly from serum, including standard cholesterol and triglyceride measures, further makes the platform more reminiscent of clinical chemistry than a technology competing with MS. However, we consider that both metabolomics technologies have great potential in epidemiologic studies and will, in many circumstances, complement each other. We anticipate more applications combining NMR and MS in the near future, particularly as cost-efficiency in MS methodologies and implementations is improved (52–54).

Although the featured NMR platform is currently the only one applied for serum metabolomics in large-scale epidemiology, alternative high-throughput NMR setups exist for detailed lipid analyses (25, 33, 55). In particular, the method for quantifying lipoprotein particle numbers offered by LabCorp (Raleigh, North Carolina) has been widely used in epidemiologic studies as well as in clinical settings (56, 57). This approach has recently been extended to cover quantification of the inflammatory biomarker glycoprotein acetylation (58, 59). This indicates a step towards combining lipoprotein profiling with quantification of nonlipid biomarkers, a feature otherwise unique to the NMR platform reviewed here. Other large-scale applications of NMR metabolomics have pertained to urine analyses (60–62) and combination of urinary and circulating metabolite data in genomic studies (63, 64), as reviewed previously (20).

## METABOLIC PROFILING IN EPIDEMIOLOGY

Comprehensive metabolic profiling has recently started to fulfill the promise of benefits for epidemiologic research (5, 11, 19). Although many metabolic measures are quantified simultaneously, the same statistical methods can be used as for other clinical chemistry assays. For instance, linear regression modeling can be applied to each metabolic measure individually. This is useful for the initial biomarker assessment and replication, because it facilitates the biological interpretability and enables adjustment for relevant confounders. The multivariate statistical analyses often applied in metabolomics studies make it difficult to integrate analyses with other risk factors and relate results to more established measures. However, analyzing the quantitative biomarker data using standard medical statistics makes it straightforward to combine analyses of the metabolic biomarker panel with conventional risk factors. This can help to put the association magnitudes of novel biomarkers into context, and it further allows investigators to extend the analyses to cover more measures than are obtained by a single metabolomics platform. Nevertheless, straightforward applications of linear regression analyses by no means preclude multivariate or nonlinear analyses—on the contrary, quantitative molecular data facilitate many different statistical approaches (5, 6, 65).

The NMR platform produces the full set of biomarkers for every sample. However, once the data are obtained, investigators can report results from individual biomarkers (e.g., omega-3 fatty acids (66)), single metabolic pathways (e.g., fatty-acid balance (34)), or all the metabolic measures from the platform (67). There are many scientific advantages in assessing the comprehensive biomarker data across multiple metabolic pathways. In addition to biomarker discovery, this approach has proven to be a powerful way to study risk factors and disease processes that have a systemic impact on the metabolic profile. For instance, adiposity broadly affects systemic metabolism, and comprehensive metabolic profiling therefore provides a more realistic view on the overall molecular associations, many of which would be missed by focusing on established risk factors or single pathways (8). In the case of risk prediction, it is also an asset to have an extensive set of metabolic biomarkers at a fixed cost—the simultaneous quantification of the routine lipid panel, glucose, and inflammation along with many other emerging biomarkers may eventually prove to be pivotal for cost-effective clinical applications (7, 13, 68).

Quantitative metabolomics data allow for hypothesis-driven and hypothesis-free research approaches both. In the case of biomarkers not previously investigated in large cohorts, the hypothesis-free approach feeds hypothesis generation—if there are no prior data on a particular biomarker, an informed hypothesis is unlikely. Regardless of the analytic approach, demonstrating replication in independent samples is important. By replication we do not mean that separate discovery and replication cohorts (as in Fischer et al. (37)) would be necessary. Rather, as recommended for genome-wide association studies (69), joint analyses of multiple independent cohorts and demonstration of consistency (as exemplified in Würtz et al. (13)) is becoming the most common approach. Regarding statistical significance, it is important to account for multiple testing whenever a high number of metabolites are tested. A significance threshold that accounts for multiple testing of correlated measures can be derived by Bonferroni correction for the number of principal components explaining 95%–99% of the variation in the metabolic data (70). For the NMR platform featured in this review, this number is typically 30–50 for each cohort, resulting in a significance threshold of *P* ≈ 0.001. However, we always advocate replication to judge the robustness of metabolic associations rather than relying on cutpoints for statistical significance.

## OVERVIEW OF LARGE-SCALE METABOLIC PROFILING STUDIES BY NMR

By mid-2016, over 400,000 blood samples from some 150 epidemiologic and clinical studies had been profiled using this NMR platform. These include the INTERVAL study, a randomized trial of blood donors with more than 46,000 individuals (71); the London Life Sciences Prospective Population (LOLIPOP) study with around 30,000 individuals (72); multiple birth cohorts and other population-based studies with several thousand participants each; and twin studies, as well as drug trials and other intervention studies (5). Many of the individual studies are working collaboratively to support replication and, where appropriate, pooling of results to obtain precise estimates and sufficient power for genetic analyses. Table 1 lists the publications to date, in which metabolic profiling data on more than 5,000 people per study have been analyzed; most of the studies include multiple cohorts and some form of replication. In more than half the studies, the number of people with metabolic profiling data is approximately 10,000 or more. The largest study published features around 35,000 samples analyzed (13). In the following section, we highlight two of these studies in more detail: 1) an evaluation of the causal effects of adiposity on systemic metabolism; and 2) an assessment of the metabolic effects of statin treatment beyond their known effects on low-density lipoprotein (LDL) cholesterol.

### Molecular characterization of adiposity

Many risk factors plausibly affect multiple molecular pathways, but the extent of this is largely unknown because of the inability to study comprehensive influences on systemic metabolism until recently. Determining the metabolic association patterns across multiple pathways can also help to pinpoint similarities in the molecular signatures of different risk factors, as in the case of body mass index (BMI) and birth weight (8, 73).

Our study on metabolic signatures of adiposity in 12,644 adolescents and young adults illustrates the metabolically diverse effects of BMI (8). BMI was robustly associated cross-sectionally with numerous metabolic biomarkers, as illustrated for selected measures in Web Figure 2. In addition to cross-sectional associations, Mendelian randomization (use of genetic instrumental variables), suggested that BMI had causal effects on multiple metabolic pathways, including atherogenic lipoproteins and lipids, fatty acids, and amino acids (Web Figure 2). The effect of adiposity on systolic blood pressure illustrates the possibility of combining the metabolic data with traditional risk markers. Having adequate power to conduct Mendelian randomization is a benefit of quantitative metabolic profiling in large cohorts (5, 74).

The overall patterns of metabolic associations were similar for cross-sectional and causal estimates. To summarize the causal influences of adiposity across the comprehensive metabolic profile, we charted all causal effect estimates against the corresponding cross-sectional associations (Web Figure 2). The close resemblance indicated that the associations between BMI and circulating biomarkers are likely to reflect the molecular effects of adiposity rather than arising from confounding or reverse causality. Although the effects of adiposity on each individual biomarker are modest, the overall metabolic aberrations may have considerable effects on cardiometabolic risk. Thus, the importance of excess adiposity likely arises from multiple metabolic pathways rather than pertaining to individual risk markers. The linear character of the metabolic associations and the Mendelian randomization analyses further suggest that there is no BMI threshold at which its adverse metabolic effects notably increase. However, analyses of metabolic changes during 6-year follow-up, in a subset of 1,466 young adults, demonstrated that the metabolic profile is highly responsive to changes in BMI with changes congruent with expectation from the results from cross-sectional and Mendelian randomization analyses (8).

### Combining metabolic profiling and genetic data for exploring drug effects

Metabolic profiling of clinical trial samples can be an important resource to assess risk prediction in specific patient groups as well as to provide improved understanding of the molecular effects of interventions. By exploiting various epidemiologic study designs, it may be possible to estimate the metabolic effects of certain drugs even when randomized evidence is not available. For instance, this can be done by examining the metabolic changes associated with starting and stopping the pharmacological treatment in longitudinal studies of observational cohorts, as we have recently demonstrated with statins (13) and hormonal contraception (75). The detailed metabolic effects can in some circumstances be assessed already at the preclinical stage based on genetic variants mimicking the pharmacological action of the drug targets (i.e., using Mendelian randomization) (30, 51, 76). This approach circumvents confounding by indication and other biases inherent in observational studies, although it may be biased by violation of the assumptions of instrumental variables (77–79). In a proof-of-concept study, we combined these 2 approaches to demonstrate how metabolic profiling in observational cohorts can be used to characterize comprehensive metabolic effects of statin therapy (13). The characteristics of the approach are shown in Web Figure 3.

Statins reduce LDL cholesterol concentration by inhibiting 3-hydroxy-3-methylglutaryl-coenzyme A reductase (HMGCR), leading to a proportionate reduction in CVD risk. Statins have been ascribed myriads of pleiotropic properties beyond lowering LDL cholesterol, yet the effects on many lipids and other biomarkers have not been assessed in large studies, primarily due to lack of affordable means. Because no randomized trial data on the metabolic biomarkers were available in our proof-of-concept study (13), the detailed metabolic effects of statins were analyzed from serially collected blood samples, in which a subset of individuals started statin therapy during follow-up. These longitudinal analyses were replicated across 4 cohorts, with consistent results despite differences in demographics and follow-up duration. Starting statin therapy was associated with changes in numerous lipid measures in addition to the anticipated lowering of LDL. Of particular interest was a discordance between the modest lowering of total triglycerides and an efficacious lowering of cholesterol in the very-low-density lipoprotein and intermediate-density lipoprotein particles (i.e., the so-called remnant cholesterol that has been identified as a potential causal culprit in the development of ischemic heart disease) (80, 81). The detailed metabolic profiling suggested that statins are more effective in reducing remnant cholesterol than previously appreciated; this indicates potential cardioprotective benefits of statins beyond LDL-cholesterol lowering. Statin use was not robustly associated with changes in any of the nonlipid metabolites assayed by the platform. These results suggest no substantial side effects of statins on, for example, circulating amino acids. However, larger studies or randomized trials are required to demonstrate potential minor effects on the nonlipid biomarkers, such as glycemic effects of statins (82).

To verify that the observed metabolic changes were actually due to the effects of statins, the analyses were corroborated via Mendelian randomization by using a genetic variant in the *HMGCR* gene as an unconfounded proxy for the pharmacological action of statins. Specifically, we examined the metabolic effects of genetically induced HMGCR inhibition—mimicking a very small statin dose—and compared the metabolic association pattern with *HMGCR* genotypes to the metabolic changes observed longitudinally. We found striking concordance between the observational effects of statins on the metabolic profile and the corresponding associations with the genetic variant in *HMGCR*.

The combination of metabolomics data with genetic data in a large number of individuals readily extends beyond studying statin effects. This type of Mendelian-randomization study design can be seen as a “natural” clinical trial (14). Due to the prohibitively high costs of randomized trials, it is of great interest to assess the detailed metabolic effects of novel targets already in preclinical stages of drug development. Many known and novel drug targets have established genetic proxies mimicking their pharmacological actions, which enables examination of the detailed metabolic association patterns of these targets. We have previously published the metabolic associations of genetic variants in the proprotein convertase subtilisin/kexin type 9 and other lipid genes (5, 51, 83). With the genome-wide association summary statistics publicly available for 123 metabolic measures (30), the fine-grained metabolic signature related to numerous genetic targets can easily be assessed. As extensive metabolomics and genetic data become increasingly available, we expect that comprehensive metabolic profiles of drug targets will augment drug development in preclinical stages to elucidate molecular mechanisms and clarify pleiotropic effects. It may be particularly helpful to use this type of approach to predict whether it would be worth moving forward to large-scale trials.

## FUTURE PROSPECTS

The studies summarized in Table 1 show some of the potential value of having quantitative metabolomics data in large epidemiologic studies. In the future, we anticipate further integration of metabolic profiling with genetics and other -omics data in large epidemiologic studies. Genome-wide studies on metabolic traits have so far primarily clarified the genetic basis of systemic metabolism (11, 30, 84, 85). The increasing collections of large-scale metabolic profiling with genetics will, via Mendelian randomization, further help to establish causality of the biomarkers as molecular intermediates between lifestyle exposures and diseases (86, 87). Detailed lipoprotein subclass profiling in combination with genetics and clinical trials are likely to be important for uncovering the mechanisms underpinning how triglyceride-rich lipoproteins relate to CVD risk (80, 88) and clarifying the elusive role of high-density lipoprotein in CVD (80, 89). With the linkage of metabolomics data to health-care records, under appropriate ethical and governance frameworks, the potential value of these new quantitative biomarkers could be explored in real-time public health applications. In the near future, alongside continued improvements in throughput and cost-effectiveness, we also look forward to endeavors of multi-omics studies on population cohorts and biobanks with over a million individuals.

## Supplementary Material

Web Material
