# Supplementary material for: Quantitative Serum Nuclear Magnetic Resonance Metabolomics in Large-Scale Epidemiology: A Primer on -Omic Technologies
Source: Am J Epidemiol. 2017 May 10;186(9):1084–96. doi: 10.1093/aje/kwx016 (PMC5860146; doi:10.1093/aje/kwx016)
Supplement: Web Material [file kwx016ala-korpelawebmaterialfinal.pdf]

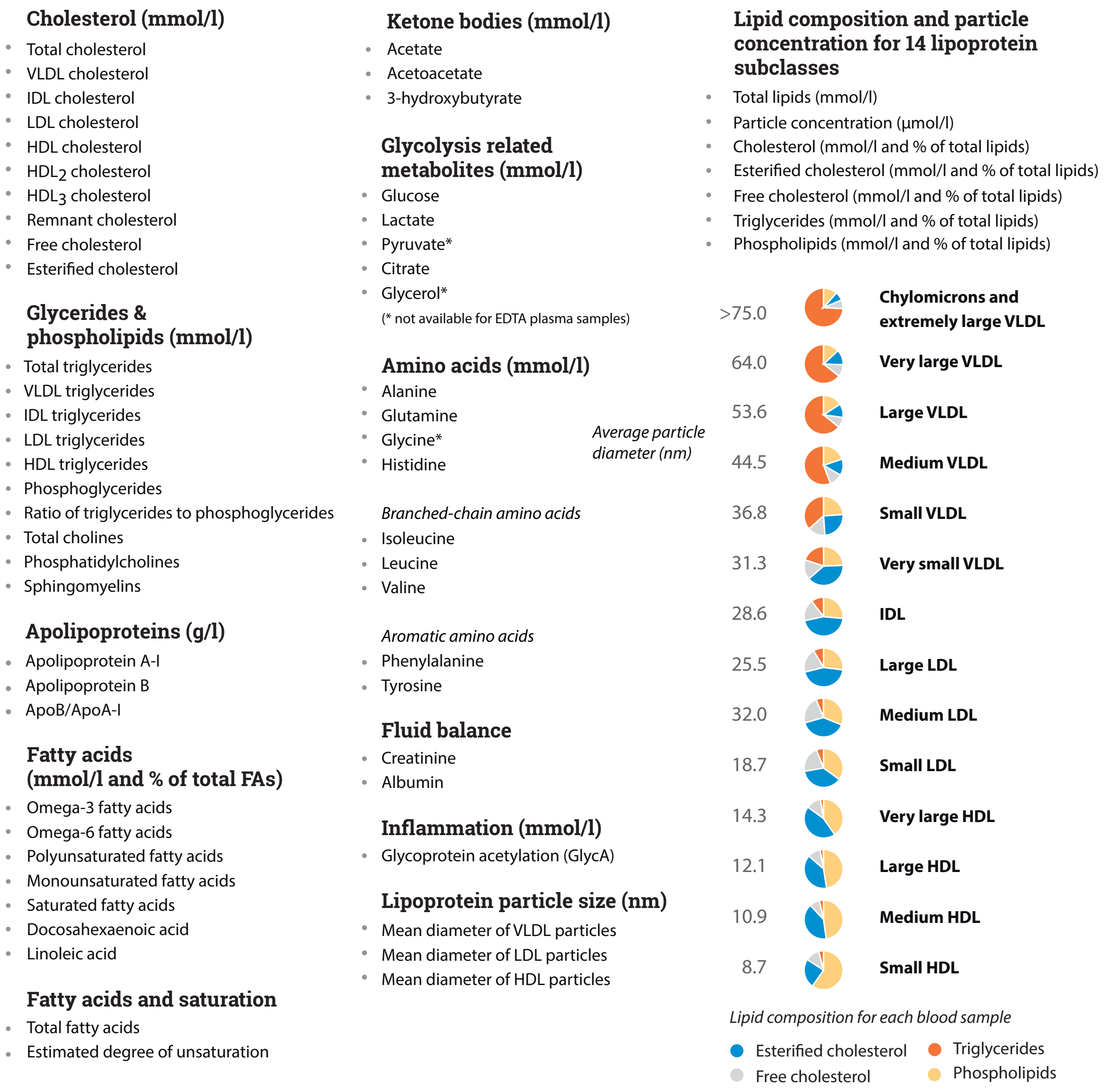

Web Figure 1. The NMR metabolomics platform quantifies 228 metabolic measures for each sample. Serum or plasma samples of either 100 or 350 μL volume can be profiled, yielding the same panel of biomarkers. Both fasting and non-fasting serum and plasma samples can be analyzed. The biomarkers are quantified in absolute concentration units, facilitating epidemiological analyses using standard medical statistics. Key categories of the metabolic measures are emphasized in the figure. For each of the 14 lipoprotein subclasses 12 measures are provided: the circulating concentration of total lipids in the particles (sum of free and esterified cholesterol, triglycerides and phospholipids), the particle concentration, and the absolute circulating concentration of five main lipids (free, esterified and total cholesterol, triglycerides and phospholipids) and the relative proportions of these

five lipids in each particle subclass. The lipoprotein subclasses are defined according to their particle size as illustrated in the figure. The metabolic measures shown here refer to the coverage of the platform as released in 2014 (5). One study (68) in Table 1 features this more extensive metabolic panel, whereas the others have used a previous smaller set of metabolic measures (39) that did not include complete lipid quantification for the 14 lipoprotein subclasses and thereby was not able to provide the lipoprotein lipid composition measures. Coefficients of variation (CV) are below 5% for more than 75% of the metabolic measures, as assessed over thousands of samples (a detailed table given in Ref. (30)). Representative CVs are: Total cholesterol 2.1%, LDL cholesterol 2.3%, HDL cholesterol 2.3%, total triglycerides 1.2%, apolipoprotein B 2.2%, apolipoprotein A-I 1.6%, omega-3 fatty acids 2.7%, omega-6 fatty acids 4.5%, proportion of omega-6 fatty acids relative to total fatty acids 2.2%, glucose 2.6%, leucine 1.9%, phenylalanine 3.9%, creatinine 3.9%, glycoprotein acetylation (GlycA) 1.1%. The CVs for lipoprotein subclass measures are generally below 5%.

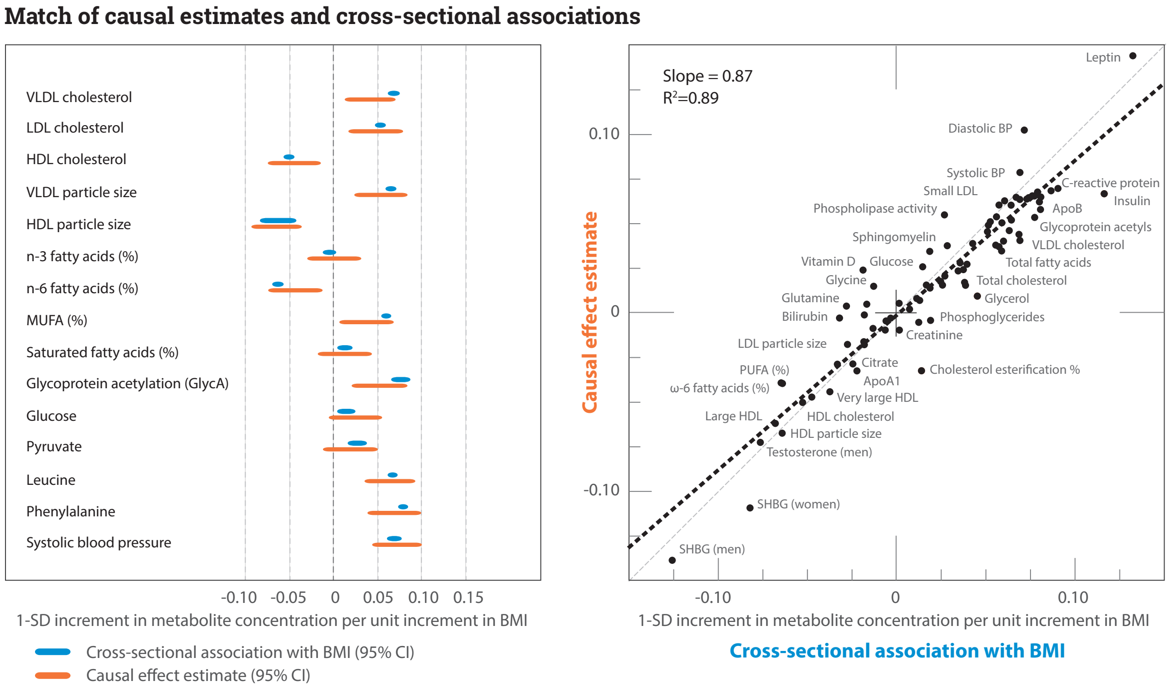

Web Figure 2. Causal effects of adiposity across the metabolic profile. Left: Cross-sectional associations (blue) and causal estimates (orange) of BMI with selected metabolic measures in 12,664 adolescents and young adults. The causal effects are based on Mendelian randomization using a gene score of 32 BMI loci as the instrument. **Right:** Consistency between cross-sectional associations and causal effects across the systemic metabolic profile. The resemblance was quantified by the R^2 and the slope of the linear fit, summarizing the causality of an exposure on multiple metabolic measures simultaneously. The figure is adapted from Reference (8).

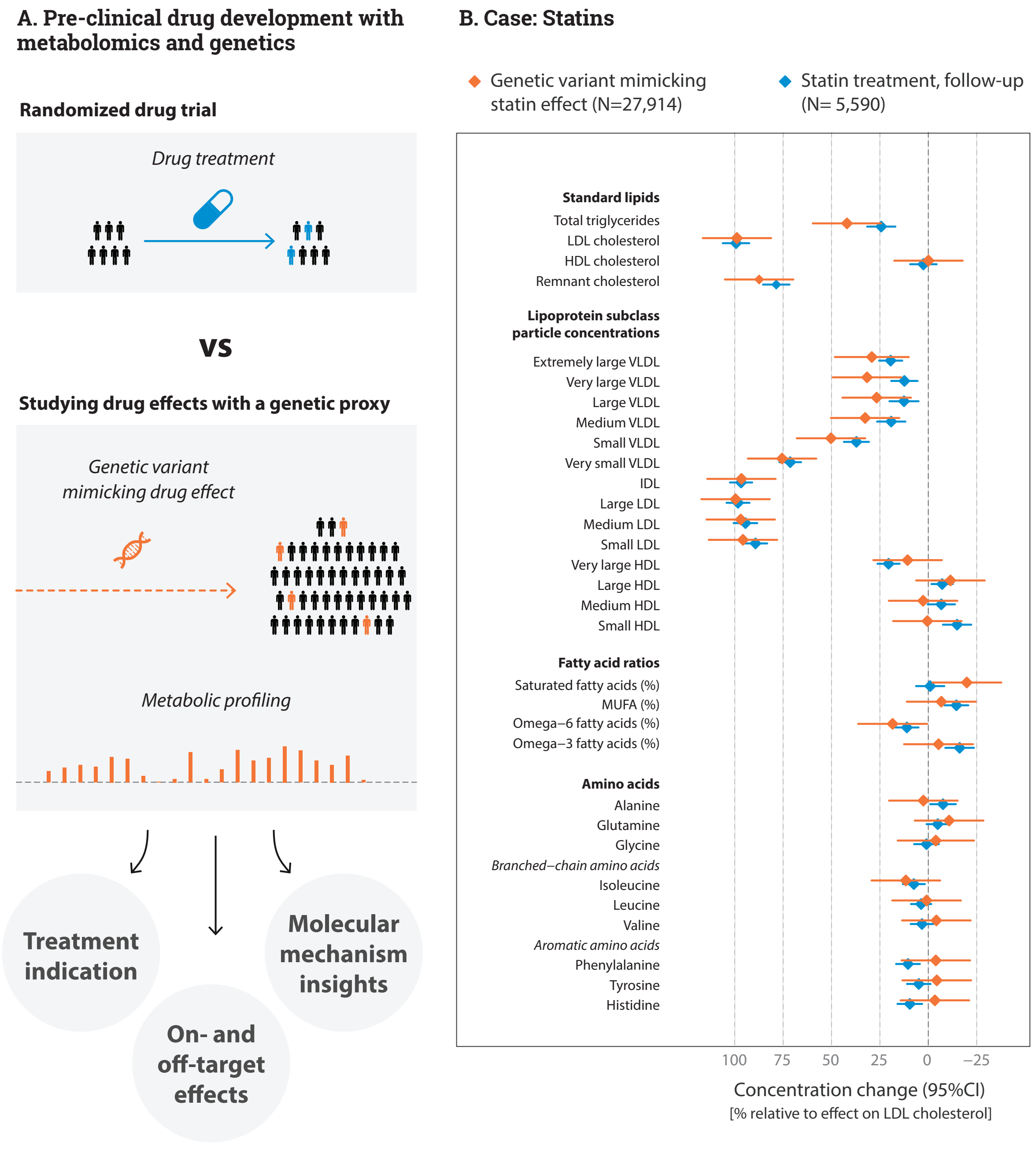

Web Figure 3. Metabolomics and genetics to study pharmacological effects pre-clinically, in trials, and in observational studies: case statins. A: Metabolic profiling of clinical trials can clarify the molecular effects of pharmacological interventions. By assessing the comprehensive metabolic associations with genetic variants that mimic the pharmacological action, the detailed metabolic effects of the drug targets can be assessed already in the pre-clinical stage (30,77). **B:** The ability of metabolic profiling to elucidate the detailed metabolic effects of known and new drug targets was recently demonstrated with statin therapy as a

proof-of-concept (13). The metabolic associations (orange bars) with a variant in the *HMGCR* gene (the intended drug target) assessed in 27,914 individuals revealed both known and novel molecular effects of statins, for instance only minimal effects on amino acids. The intricate metabolic signature of *HMGCR* was almost exactly recapitulated in the detailed metabolic changes associated with statin therapy during follow-up in four longitudinal cohorts (blue bars). Both genetic and longitudinal evidence indicated that statins are efficacious at lowering remnant cholesterol levels, among several other lipid effects extending beyond LDL cholesterol. The figure is adapted from Reference (13).
